# Fabrication of Ordered Mullite Nanowhisker Array with Surface Enhanced Raman Scattering Effect

**DOI:** 10.1038/srep09690

**Published:** 2015-04-13

**Authors:** Tao Yang, Enhui Wang, Fuqiang Wang, Kuochih Chou, Xinmei Hou

**Affiliations:** 1 State Key Laboratory of Advanced Metallurgy, University of Science and Technology Beijing, Beijing 100083, China

## Abstract

Mullite nanowhiskers are prepared by a facile technique at low temperature using mica and AlF_3_ as raw material. Mica acts as reactant as well as substrate. By controlling the reaction temperature and holding time, the mullite nanowhisker array with uniform morphology is obtained. The nanowhisker array possesses Al-rich single crystalline with an average of 80 nm in diameter and 20 μm in length. After decorated with Au nanoparticles, the array exhibits high surface enhanced Raman scattering (SERS) activity with an SERS enhancement factor (*EF*) of 1.35 × 10^9^. It also remains good SERS signal detection with a relative standard deviation of 7.33% under corrosion condition.

Surface enhanced Raman scattering (SERS) technique is one of the most powerful analytical tools for chemical and biological detection due to its high sensitivity and specificity^1^,^2^,^3^,^4^. Since its emergence in the 1970s, SERS has been a hot topic due to its nondestructive nature, ultrahigh sensitivity and the richness of molecular information offered^5^,^6^,^7^,^8^,^9^,^10^,^11^. Generally noble metal nanostructures, especially Au and Ag have excellent SERS activity and thus are conventional SERS substrates used for the ultra-sensitive detection^12^,^13^,^14^,^15^. However the high cost and complex synthesis process of Au and Ag substrates restrict their wide application^16^,^17^,^18^,^19^,^20^. Preparation of SERS metallic array nanostructures with high sensitivity in a simple method remains a challenge for routine SERS detection.

Mullite as an engineering material has been widely used in the last decades because of its good mechanical strength, excellent thermal shock and high creep resistance, low thermal conductivity and good stability under corrosion conditon^21^. Recently mullite nanowires have been reported using various methods such as sol-gel^22^,^23^, high-energy ball milling process^24^,^25^,^26^, thermal decomposition of minerals and molten salt synthesis (MSS)^27^,^28^,^29^ *etc.*. This will absolutely extend its application field. Considering mullite is very stable even at acid and alkali atmosphere^21^, it offers us a hint that mullite nanowires can be adopted as the SERS substrate used under corrosion condition. Aiming at enhancing the detection sensitivity, it requires the morphology of the substrate should be highly ordered along one direction to get good repeatability and at the same time has “hot spots” as many as possible. Therefore mullite nanowires with highly ordered structure are primarily requirement as SERS substrate. Mullite nanowhiskers with Al-rich structure have been synthesized using various techniques^22^,^23^,^24^,^25^,^26^,^27^,^28^,^29^. But these researches mainly focus on preparation of well disperse mullite whiskers. In addition, the morphology of mullite is difficult to be tuned due to the complicate reaction condition. Up to now, there is no report about synthesis of mullite array with uniform morphology. Mica is widely applied in electronic industry as a kind of natural minerals. The main compositions of it are Al_2_O_3_ and SiO_2_, which are also the composition of mullite^21^. In addition, mica can be easily processed into flaky shape because of its crystal character^30^. Therefore it can be adopted as raw material as well as substrate to produce mullite whisker array.

Herein in this work, we design a facile and economical method to obtain mullite nanowhisker array from mica as shown in Figure 1. Mica acts as reactant as well as substrate. The morphology of mullite nanowhisker array is tuned by simply changing the temperature and holding time. Then the array decorated with Au nanoparticles is applied as SERS substrate. It exhibits high sensitivity in SERS detection of a target analyte, Rhodamine B (RhB). The SERS signal remains high sensitive and stable with a relative deviation of 7.33% under corrosion condition.

## Results

### Fabrication of mullite nanowhisker array

**Figure 2:**
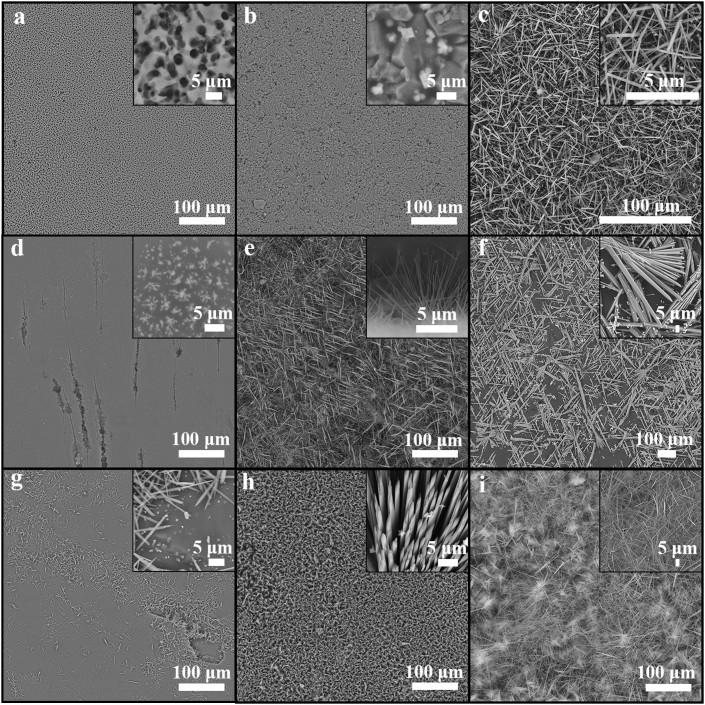
SEM micrographs of the obtained samples. (a)–(c) were calcined at 900°C for 1 h using different substrates ((a) quartz, (b) corundum, (c) mica). (d)–(f) were calcined at different temperature for 1 h using mica as substrate((d) 800°C, (e) 900°C, (f) 1000°C). (g)–(i) were calcined at 900°C for different time using mica as substrate((d) 1 h, (e) 3 h, (f) 5 h).

The key factors affecting the morphology of mullite nanowhiskers array were studied. Fig. 2 shows SEM micrograph of the sample obtained under different condition. All the experiments were carried out in static air. Figs. 2a–c show the effect of substrate on mullite formation. The experiments were carried out at 900°C for 1 h using quartz, corundum, mica as the substrate respectively. Obviously, mullite nanowhiskers are synthesized using mica with the size of 20 × 10 mm as substrate (Fig. 2c). Figs. 2d–f present the morphology of mullite obtained at different temperature i.e. 800, 900 and 1000°C. The samples were obtained at the required temperature for 1 h using mica as the substrate. Mullite whiskers with small amount are obtained at 800°C (Fig. 2d). When the temperature increasing to 900°C, the morphology of mullite becomes uniform and the amount is larger (Fig. 2e). While at 1000°C, mullite whiskers with irregular morphology are obtained due to aggressive crystal growth (Fig. 2f). Figs. 2g–i indicate the effect of time on the morphology of mullite. The samples were obtained at 900°C for 1, 3 and 5 h using mica as the substrate. Because of the short holding time, i.e. 1 h, only a small amount of mullite whiskers are observed on the surface of mica (Fig. 2g). In addition, the length of the whisker is relatively shorter. It can be seen from Fig. 2h that large scale mullite whiskers with a preferred orientation are synthesized at 900°C for 3 h. With time further prolonging, mullite whiskers become so long that the whiskers fall down (Fig. 2i). From above experimental results, large scale mullite whisker array with uniform morphology is synthesized at 900°C for 3 h in static air using mica as substrate.

### Characterization

**Figure 3:**
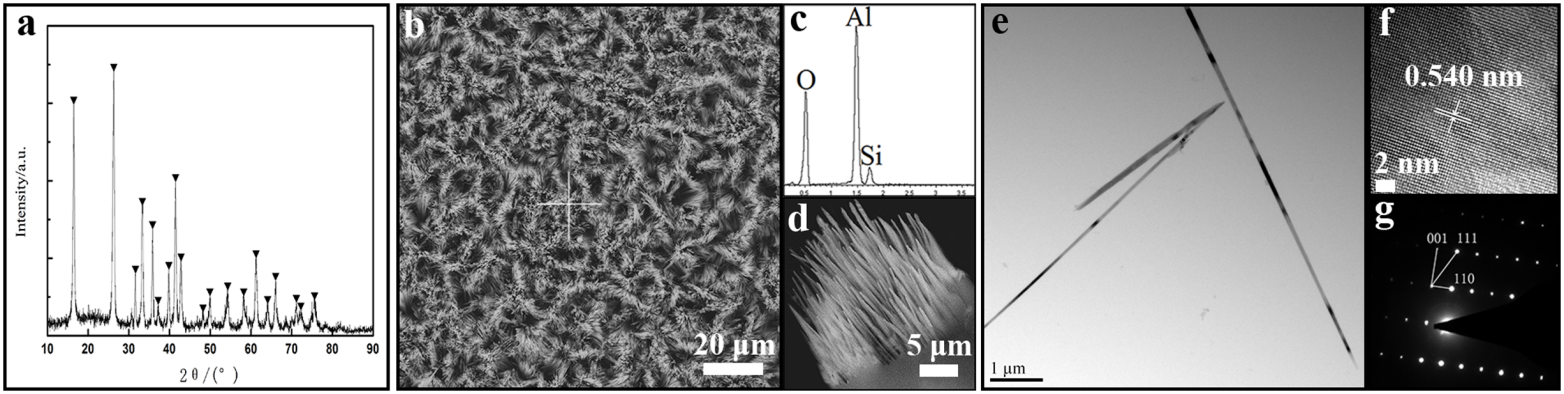
XRD pattern, SEM and TEM micrographs of the mullite nanowhisker array obtained at 900°C for 3 h using mica as substrate. (a) XRD pattern of the obtained mullite nanowhisker. (b)(d) SEM micrographs of the obtained mullite nanowhisker array. (c) The EDS spectra of the obtained mullite nanowhisker. (e) TEM micrographs of the mullite nanowhiskers at low magnification. (f) HRTEM. (g) The SAED pattern.

Fig. 3a shows XRD patterns of the sample obtained at 900°C for 3 h in static air. It shows that only mullite phase is detected. The characteristic peaks can be indexed to the orthorhombic structure, i.e. Al_2_(Al_2.8_Si_1.2_)O_9.6_ (PDF#79-1275), which is Al-rich phase. The microstructure characterization of the obtained product is shown in Fig. 3b–g. It can be seen from low magnification of SEM image (Fig. 3b) that large scale mullite whiskers with a preferred orientation are synthesized. From the side view (Fig. 3d), the whiskers grown on the substrate are self-assembled into aligned array with the average length of 20 μm. EDS spectra (Fig. 3c) reveal that the whiskers are composed of Al, Si and O. Further microstructure carried out by TEM (Fig. 3e) indicates the mullite whiskers have a smooth and clean surface with the average diameter of 80 nm. The high resolution TEM (HRTEM) images shown in Fig. 3f reflect that the whiskers are single crystalline and the lattice fringes are well defined. The interplanar spacings of 0.540 nm detected from the legible lattice fringes along the axis of the whisker is quite similar to that of the (110) planes of Al_2_(Al_2.8_Si_1.2_)O_9.6_, indicating that the preferential growth direction of the nanowhiskers is parallel to the (110) planes. The corresponding selected area electron diffraction (SAED) pattern (Fig. 3g) can be indexed to (110) and (001) planes respectively, further verifying the preferential growth direction of the nanowhiskers is parallel to the (110) plane.

From above experiment, the vapor-solid (VS) mechanism is proposed for the formation of mullite nanowhisker array because the ends of the whiskers are facets without droplet existing (Fig. 3d)^31^. During the whole process, the intermediate fluorides play a critical role for mullite crystal growth^32^,^33^. In the experiment, fluoride vapor such as AlOF is produced by the reaction of AlF_3_ with the oxygen in the chamber (Eq. (1)) at high temperature. AlOF is transferred to the mica substrate located above AlF_3_ zone (approximately 1–2 cm). When the vapor reaches the mica, it reacts with mica resulting in continuous release of AlOF and SiF_4_ vapor (Eq. (2)). The relatively closed environment in this experiment leads to a small vapor partial pressure and supersaturation of mullite nuclei. This promotes the growth of 1D mullite nanowhiskers rather than 3D particles (Eq. (3)). Since mica adopted in the experiment acts as substrate at the same time, the mullite nanowhisker array is formed.
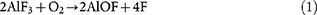

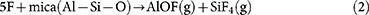

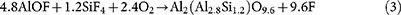


### SERS activities of mullite nanowhiskers array

**Figure 4:**
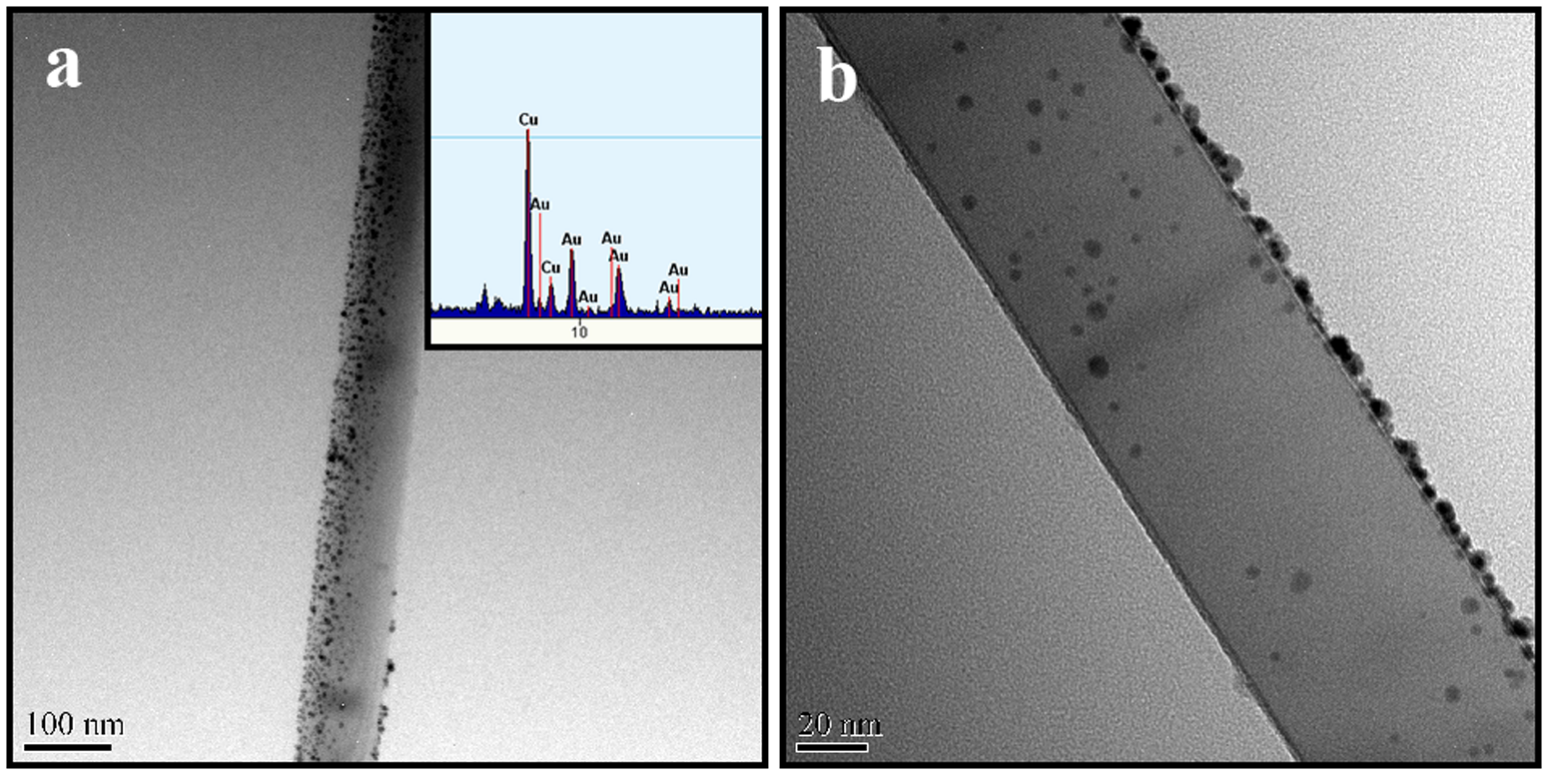
TEM micrographs of the mullite nanowhisker array covered with Au nanoparticles.

The possibility application of mullite nanowhisker array for SERS detection is investigated. Au particles coated on the mullite nanowhisker array is obtained using physical vapor deposition (PVD). The sample is sputtered coating for 6 min using the sputter ion plating apparatus at the lowest voltage to control the gold particle size within 10 nm. The microstructure of the mullite nanowhisker array coated with gold particles is examined by TEM (Fig. 4). It clearly indicates that large quantity of small particles with the size of 5–10 nm is assembled onto the surface of the mullite whiskers (Fig. 4a and b). The EDS spectra (inset Fig. 4a) reveal that the particles on the surface of mullite nanowhiskers are Au particles.

**Figure 5:**
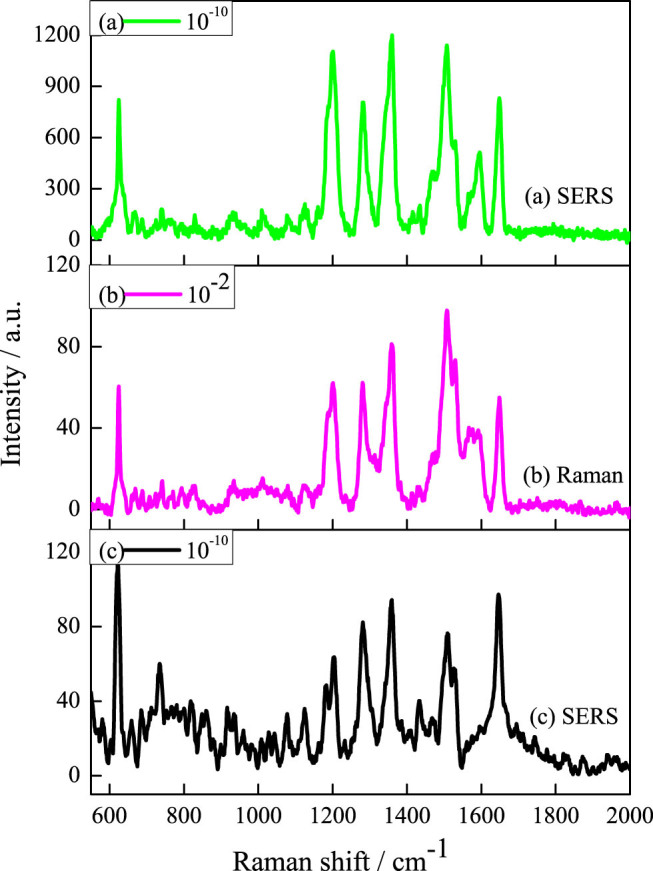
SERS spectra acquired from RhB adsorbed on the different substrates. (a) The mullite nanowhisker array covered with Au particles. (b) Only the mullite nanowhisker array. (c) The pure mica substrate deposited with Au nanoparticle. All of the spectra are acquired at an excitation of 632.8 nm.

Rhodamine B (RhB) is taken as the probe molecule excited by the laser of 632.8 nm. It is reported that the characteristic peaks of RhB at 423, 620, 765, 933, 1201, 1280, 1356, 1433, 1506, 1529, 1565, 1595 and 1648 cm^−1^ are corresponding to the stretching vibrations of RhB bands^11^,^34^. The signals between 1306 and 1650 cm^−1^ are attributed to atomatic C-C stretching vibrations^35^,^36^,^37^. Fig. 5a shows the SERS spectra of RhB (10^−10^ M) on the mullite nanowhisker array coated with gold particles.

For comparison, the bulk Raman spectra of 10^−2^ M RhB on the mullite nanowhisker array (Fig. 5b) and SERS spectra of RhB (10^−10^ M) using a pure mica substrate deposited with Au nanoparticle under the same condition (Fig. 5c) are also detected. Although the typical SERS spectrum of RhB with plenty of peaks can be seen using the three substrates. Especially the predominant bands located at 624, 1203, 1281, 1359, 1507, 1530, 1596 and 1648 cm^−1^ in the SERS spectrum are enhanced with inappreciable frequency shifts compared with the literature report. However the predominant bands in the SERS spectra of RhB (10^−10^ M) on the mullite nanowhisker array coated with gold particles are highly enhanced. There are also several weak Raman shifts have no accurate assignments, such as the Raman shifts of 933, 765 and 400 cm^−1^.

**Figure 6:**
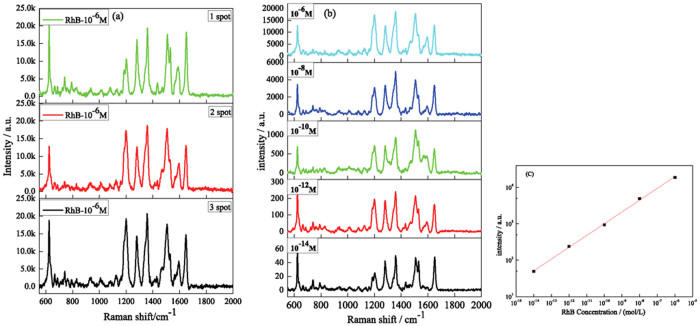
(a) Repeatability of SERS spectra of RhB adsorbed on the mullite nanowhisker array coated with Au nanoparticles. (b) Concentration-dependent SERS spectra of RhB taken from Au-covered mullite nanowhisker array substrate. (c) A semi-log plot of the concentrationversus 1359 cm^−1^ peak intensity.

3 spots are randomly chosen and measured. The concentration of RhB is 10^−6^ M. It can be seen that all the characteristic peaks of RhB are detected and the intensity of the peaks is similar as shown in Fig. 6a, verifying the reasonability of the SERS measurement. To quantify the SERS activity of the mullite nanowhisker array, *EF* was calculated by comparing the intensity of a single molecule from the SERS signal with that from the bulk Raman signal by the formula^11^ as follows:
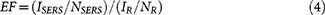
where *I_SERS_* and *I_R_* are the intensities of the selected scattering bands in the SERS and bulk Raman spectra, respectively, *N_SERS_* is the number of probe molecules contributing to the SERS signal, and *N_R_* is the number of probe molecules contributing to the bulk Raman signal. Assuming that the probed molecules are distributed on the substrate uniformly^11^, the number of probe molecules contributing to the signal *N* can be estimated by 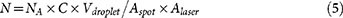
where *N_A_* is the Avogadro's number, *C* is the concentration of the used RhB, *V_droplet_* is the volume of the RhB droplet, *A_spot_* is the area of the spot formed by the RhB droplet, and *A_laser_* is the area of the laser spot. Since the specimens for SERS and bulk Raman tests are prepared in the same way and measured with the same parameters (seeing the methods), equation (4) can be written as
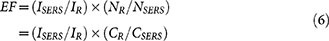


Where *C_R_* and *C_SERS_* are the concentrations of the RhB on the mullite nanowhisker array and the array coated with Au particles, respectively. Taking the two strongest peaks of 1359 cm^−1^ and 1507 cm^−1^ into account, the relevant data required by equation (6) are listed in Table 1. The *EF* is calculated to be about 1.5 × 10^9^ and 1.2 × 10^9^ by using the peaks of 1359 cm^−1^ and 1507 cm^−1^, respectively. The average *EF* is 1.35 × 10^9^, which is 1–4 orders of magnitudes higher than that of the reported the LoBs@Ag(6.9 × 10^8^)^9^, metallic glassy nanowire arrays(1.1 × 10^5^)^11^ and transparent free-standing metamaterials(8.6 × 10^6^)^38^. It indicates that the nanostructures of mullite nanowhisker array can exhibit strong SERS *EF.*

To further study the SERS property of the mullite nanowhisker array, a series of SERS spectra by varying the concentration of RhB from 1 × 10^−6^ to 1 × 10^−14^ M on the mullite nanowhisker array coated with Au particles are measured as shown in Fig. 6b. The signals are found to be quite alike with almost no frequency shift. Even at a concentration as low as 10^−14^ M, the spectral features of RhB can be still seen clearly. The signals are found to be monotonically decreasing with the decreased concentration as shown in Fig. 6c. By applying the Lorentz fitting, the intensities of peaks around 1359 cm^−1^ are measured. The relevant data are listed in Supplementary Table S1. It is found that the SERS intensities (*I_SERS_*) and the concentration of RhB (*C_RhB_*) have a correlation from 10^−6^ M to 10^−14^ M as follow:
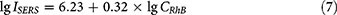
with an *R* square high than 0.997 (Fig. 6c). It indicates that the mullite nanowhisker array coated with Au particles is very suitable to be applied as quantitative sensor for chemical and biological molecules such as RhB.

## Discussion

As can be seen from the SERS spectra in Figs. 5 and 6, the mullite nanowhisker array coated with Au particles is sensitive as a quantitative sensor for chemical and biological molecules detection such as RhB. There are two major mechanisms, i.e. long-range electromagnetic (EM) enhancement and short-range chemical enhancement to explain the SERS activity^39^. In this work, the obtained EF shows that the Raman enhancement factor reaches 10^9^, which is far more than the average enhancement factor 10^2^ of chemical enhancement. It can be concluded that the electromagnetic enhancement is mainly the reason. At the same time, it can be seen that all RhB characteristic peaks are enhanced except the peak at 933, 765 and 400 cm^−1^ as shown in Figs. 5 and 6. This is a typical feature of the chemical enhancement, i.e. the characteristic peaks of probe molecules are selectively enhanced or restricted^39^,^40^. Therefore the SERS in the experiment are generated by the combination of electromagnetic enhancement and chemical enhancement. Supplementary Fig. S2a illustrates the mullite nanoarrays coated with Au particles. It has been exhibited that the mullite nanowhisker array is Al-rich structure and thus possess lots of oxygen vacancy. At the same time the sizes of the gold particles using physical vapor deposition (PVD) are in the range of 5–10 nm (Fig. 4). The small gaps between the two gold particles and the corner of mullite nanowhiskers generate a lot of “hot spots”^41^. Moreover the obtained mullite nanowhisker array possesses Al-rich structure and thus leads to the valance state of the mullite surface to be negative. The positive charged gold particles are assembled on the surface of the negatively charged mullite whiskers via the electrostatic interaction. Due to the electrostatic attraction of the negative charges of the mullite whiskers, the electron density may move toward the bottom of gold particles and generate a dipole in the particles as shown in Supplementary Fig. S2b. Since RhB molecular contains positively charged N^+^, which is easily adsorbed at the top part of Au particles. Therefore it is the changes in the static polarizability of the molecule that lead to the overall chemical enhancement.

### Applications in corrosion condition

**Figure 7:**
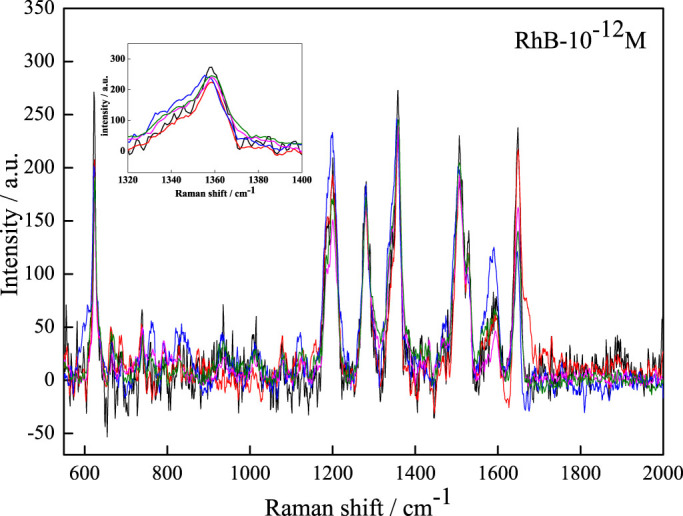
SERS spectra of 10^−12^ M RhB from 5 randomly selected positions on the Au-covered mullite nanowhisker array substrate used under corrosion condition. The inset is the enlarged detail of the peaks around 1359 cm^−1^.

Considering mullite is very stable even at acid and alkali atmosphere, mullite nanowhisker array can be used as SERS substrate applied under corrosion condition. In the experiment, mullite nanowhisker array coated with Au particles was immersed in 10^−12^ M RhB solution with 2 ml HCl (20%) added for 30 min and then rinsed by deionized water thoroughly. Fig. 7 shows the SERS spectra of 10^−12^ M RhB collected from 5 randomly selected positions on the mullite nanowhisker array coated with Au particles. The SERS signal intensity is quite uniform except few points. The relative standard deviation (RSD) of SERS peak around 1359 cm^−1^ is used to estimate the stability of the SERS signals. After fitted by the Lorenz curve, the intensities of the peaks around 1359 cm^−1^ are shown in Supplementary Fig. S1. The RSD is calculated to be 7.33%, verifying the good SERS signal detection under corrosion condition.

## Conclusions

A facile method is adopted in the work to prepare mullite nanowhisker array using mica and AlF_3_ as raw materials at 900°C for 3 h in static air. The obtained mullite nanowiskers are Al-rich single crystalline and possess uniform morphology with an average of 80 nm in diameter and 20 μm in length. The obtained mullite nanoarrys decorated by gold nanoparticles exhibit high sensitivity in detection of the target analyte, RhB with a detection limit up to 10^−14^ M. It still exhibits good SERS signal detection under corrosion condition with a relative standard deviation of 7.33%. The array exhibits high SERS activity with an EF of 1.35 × 10^9^ by the combination of electromagnetic and chemical enhancement. The present result provides insights into the application of mullite nanowhisker array for SERS detection even used under corrosion condition.

## Methods

### Materials

The chemical composition of mica used in the experiment was showed in Supplementary Table S2. It can be seen that the ratio of Al/Si in the mica was lower than that of mullite. Therefore aluminum fluoride (AlF_3_,> 99 wt %) powder was employed as additional aluminum source.

### Preparation of mullite nanowhisker array

The experiment was performed in a conventional electric furnace. Firstly an alumina boat was loaded with ~0.2 g AlF_3_ powder as raw material and one piece of quartz, corundum, mica substrate (1 cm × 2 cm) was placed right above AlF_3_ about 1–2 cm (Supplementary Fig. S3.). Then the material was placed at the hot zone of the electric furnace and was heated rapidly to 800–1000°C in a static air atmosphere for 1–5 h. Finally the furnace was cooled down naturally to room temperature. A thin layer of white products was obtained on the surface of mica substrate.

### Preparation of SERS substrate

The mullite nanowhisker array obtained above was coated with Au particles by physical vapor deposition (PVD). The sample was sputtered coating for 6 min using the sputter ion plating apparatus (Quorum/Emitech K550X) at the lowest voltage to control the gold particle size within 10 nm. These gold coated substrates were thoroughly cleaned and stored at nitrogen atmosphere.

### Characterizaition

The microstructures of the mullite nanowhisker array were examined by scanning electron microscopy (SEM, FEI Nova 230 Nano), Transmission electron microscopy (TEM, Tecnai G2 F30 S-TWIN) and Rigaku D/max-RB XRD respectively.

### SERS measurement

RhB with analytically purity was used as the probe molecule to study the SERS activities of the mullite nanowhisker array. Before the SERS measurement, a 2 mL droplet of the RhB aqueous solution was dropped on each of the mullite nanowhisker array substrates which were subsequently dried at 50°C for 30 min. The solution diffused on the surface to be a spot with 0.4 cm in diameter. The SERS measurements were carried out in the center of the droplet at room temperature by a microscopic confocal Raman spectrometer(RM2000, Renishaw PLC, England) using a charge-coupled device (CCD) detector with a resolution of 1 cm^−1^. The laser beam power was 4.7 mW and the laser beam diameter was 5 mm. Excitation wavelength of 632.8 nm (according to the previous literature^11^), scan time of 30 s, field lens of 20 times and accumulation of 4 times were applied. The SERS mapping measurements were carried out in the center of the droplet by a microscopic confocal Raman spectrometer (LabRAM HR Evolution, HORIBA Jobin Yvon, France) using a charge-coupled device (CCD) detector with a resolution of 0.65 cm^−1^.

## Supplementary Material

Supplementary Information Supplementary Information

## Figures and Tables

**Figure 1:**
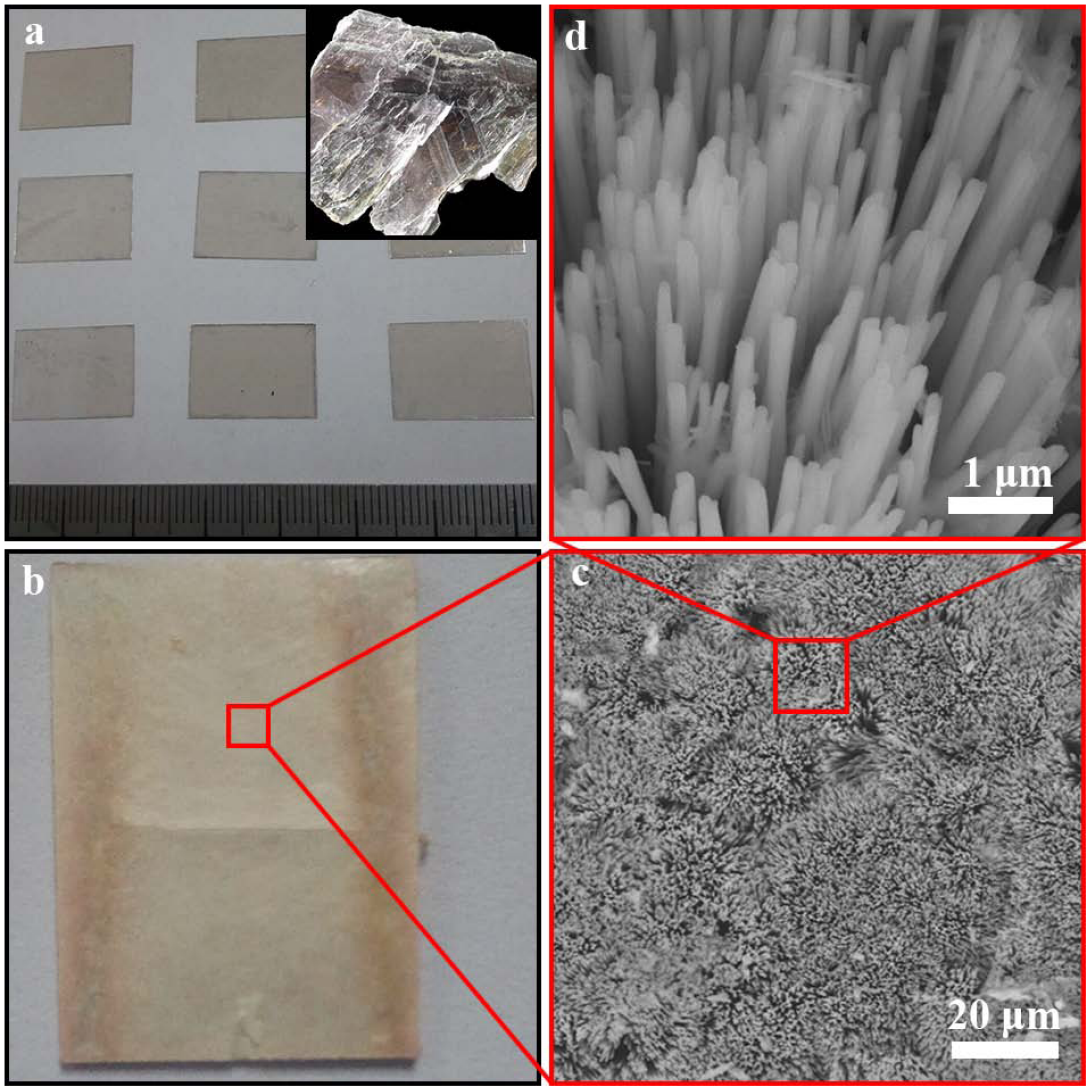
A facile method to produce mullite nanowhisker array from mica. (a) Mica substrate. Inset mica ore. (b) Mullite nanowhisker arrays on the surface of mica. (c)(d) SEM image of mullite nanowhisker arrays.

**Table 1:** Parameters for the calculation of *EF*

		Relative intensity (a.u.)		*EF*	
Spectra	C(mol·L^−1^)	1359 cm^−1^	1507 cm^−1^	1359 cm^−1^	1507 cm^−1^
SERS	10^−10^	1199	1140	1.5*10^9^	1.2*10^9^
Raman	10^−2^	81	98		
