# Supplementary material for: Fabrication of Ordered Mullite Nanowhisker Array with Surface Enhanced Raman Scattering Effect
Source: Sci Rep. 2015 Apr 13;5:9690. doi: 10.1038/srep09690 (PMC5381749; doi:10.1038/srep09690)
Supplement: Supplementary Information [file srep09690-s1.pdf]

Supplementary Information

Fabrication of Ordered Mullite Nanowhisker Array with Surface Enhanced Raman Scattering Effect

Tao Yang, Enhui Wang, Fuqiang Wang, Kuochih Chou & Xinmei Hou*

State Key Laboratory of Advanced Metallurgy, University of Science and Technology Beijing, Beijing 100083, China

Fax: +86 10 6233 2570; Tel: +86 10 6233 2570; Email: houxinmei@ustb.edu.cn

Table S1. The SERS signal intensities of the peaks of 1359 cm^{-1} using RhB with concentration from 10^{-6}M to 10^{-14}M.

concentration ($mol{\cdot}L^{-1}$)	intensity (counts)
10^{-6}	18760±110
10^{-8}	4907±65
10^{-10}	948±23
10^{-12}	241±12
10^{-14}	52±8

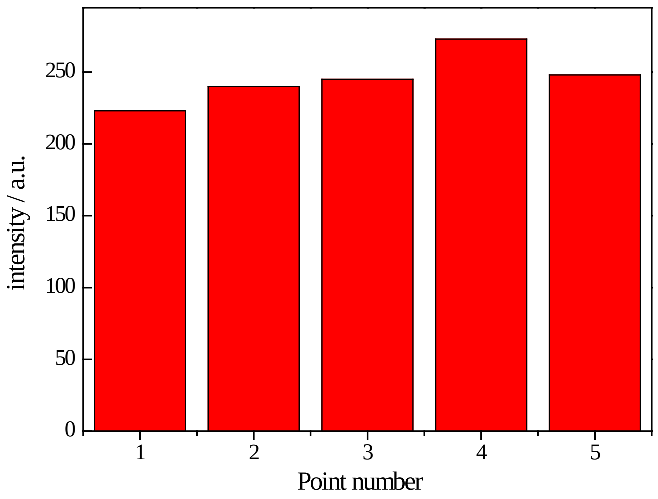

Figure [S1] Intensities of the peaks around 1359 cm^{-1} in the SERS spectra of 10^{-12}M RhB from 5 randomly selected positions on the Au-covered mullite array used under corrosion condition.

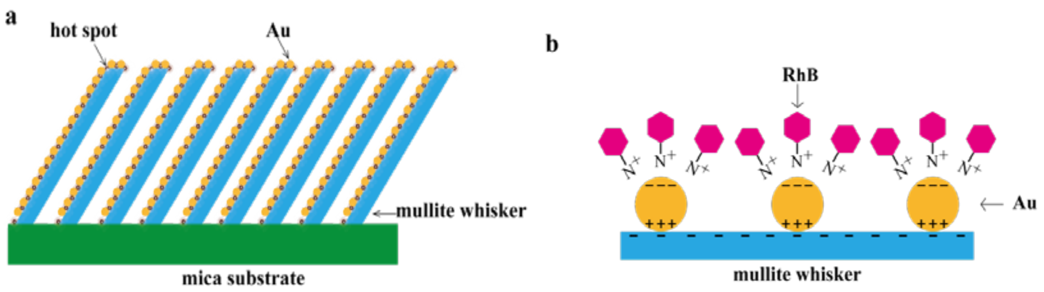

Figure [S2]. the schematic illustration of enhanced of SERS mechanisms of mullite nanowhisker array decorated with Au nanoparticles.

Table S2. the chemical composition of the mica substrate (wt%)

**Composition**	**SiO_2**	**Al_2O_3**	**Na_2O**	**Fe_2O_3**	**TiO_2**	**K_2O**	**Other**
**Mica**	46.51	30.52	1.03	3.62	0.4	14.63	3.29

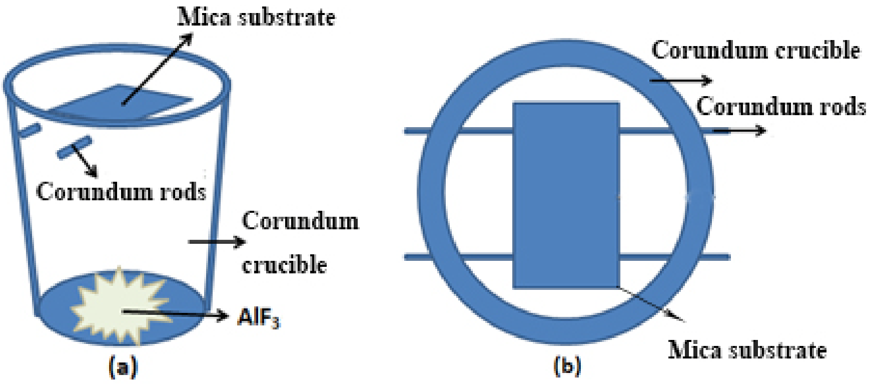

Figure [S3]. Structure of the experiment platform
